# Supplementary material for: Amplified fluorogenic immunoassay for early diagnosis and monitoring of Alzheimer’s disease from tear fluid
Source: Nat Commun. 2023 Dec 9;14:8153. doi: 10.1038/s41467-023-43995-5 (PMC10710446; doi:10.1038/s41467-023-43995-5)
Supplement: Supplementary file 1 — Supplementary Information [file 41467_2023_43995_MOESM1_ESM.pdf]

Supplementary Information

Amplified fluorogenic immunoassay for early diagnosis and monitoring of Alzheimer's disease from tear fluid

Sojeong Lee[1], Eunjung Kim[2,3], Chae-Eun Moon[4], Chaewon Park[1], Jong-Woo Lim[1], Minseok Baek[5], Moo-Kwang Shin[1], Jisun Ki[1], Hanna Cho[6,], Yong Woo Ji[4,*], and Seungjoo Haam[1,*]*

S. Lee, C. Park, J.-W. Lim, M.-K. Shin, J. Ki, and S. Haam
[1]Department of Chemical and Biomolecular Engineering, Yonsei University, Seoul 03722, Republic of Korea

Prof. E. Kim
[2]Division of Bioengineering, Incheon National University, Incheon 22012, Republic of Korea
[3]Department of Bioengineering & Nano-bioengineering, Research Center for Bio Materials and Process Development, Incheon National University, Incheon 22012, Republic of Korea

Dr. C.-E. Moon and Prof. Y. W. Ji
[4]Department of Ophthalmology, Yongin Severance Hospital, Yonsei University College of Medicine, Yongin 16995, Republic of Korea

Prof. M. Baek
[5]Department of Neurology, Wonju Severance Christian Hospital, Yonsei University Wonju College of Medicine, Wonju 26426, Republic of Korea

Prof. H. Cho
[6]Department of Neurology, Gangnam Severance Hospital, Yonsei University College of Medicine, Seoul 06273, Republic of Korea

***Corresponding Authors**
Seungjoo Haam*
E-mail: haam@yonsei.ac.kr
Yong Woo Ji*
E-mail: LUSITA30@yuhs.ac
Hanna Cho*
E-mail: IGUHANNA@yuhs.ac

Inventory of Supporting Information

Supplementary Figures

Supplementary Figure 1. Characterization of the synthesized antibody-immobilized magnetic nanoparticles (Ab-MNPs).

Supplementary Figure 2. Elemental mapping images of Ab-MNPs investigated by TEM equipped with an energy-dispersive X-ray spectroscopy detector.

Supplementary Figure 3. Magnetization curves of the MNPs series using vibrating sample magnetometry.

Supplementary Figure 4. Preparation of block copolymers.

Supplementary Figure 5. Synthetic process and characterization of antibody-immobilized polymeric nanoprobes (Ab-PNPs).

Supplementary Figure 6. Examination of the loading amount of Förster resonance energy transfer (FRET) dye.

Supplementary Figure 7. Investigation of optimal FRET dye ratios incorporated in PNPs.

Supplementary Figure 8. Examination of the reaction time required to induce the maximum occurrence of the high FRET to low FRET transition by PNP degradation with the addition of Triton X-100 as lysis buffer.

Supplementary Figure 9. Optimization of FRET signal changes of PNPs at various buffer conditions.

Supplementary Figure 10. Protein marker detection efficiency of self-assembled nanoparticle-mediated amplified fluorogenic immunoassay (SNAFIA) in tear fluid.

Supplementary Figure 11. ELISA and hSNAFIA tests with different concentrations of cyclase-associated protein 1 (CAP1) protein ranging from 0 to 25000 nM.

Supplementary Figure 12. Schematic of half–SNAFIA (hSNAFIA) designed by replacing Ab-PNPs in SNAFIA with AlexaFluor®488-conjugated antibodies.

Supplementary Figure 13. Evaluation of the SNAFIA platform toward apolipoprotein E

(APOE) spiked in human serum.

Supplementary Figure 14. Assessment of the colloidal stability of PNPs in human serum.

Supplementary Figure 15. ELISA and hSNAFIA test with different concentrations of APOE protein ranging from 0 to 2000 nM.

Supplementary Figure 16. Sensitivity test of SNAFIA with increasing concentration of CAP1 protein.

Supplementary Figure 17. Sensitivity test of SNAFIA with increasing concentration of APOE protein.

Supplementary Figure 18. Investigation of the optimal reaction time for SNAFIA tests.

Supplementary Figure 19. Correlation between the fluorescence intensity of SNAFIA obtained from a clinical tear sample (patient ID: 15–29) and the Mini-Mental State Examination score of the same subject.

Supplementary Tables

Supplementary Table 1. Demographics of participants of the discovery cohort for tear protein biomarker profiling in this study.

Supplementary Table 2. Differentially expressed proteins in tear fluid of MCI and AD compared to HC.

Supplementary Table 3. Size of MNPs, SiMNPs, CMNPs, and Ab-MNPs estimated by DLS and TEM image-based analysis.

Supplementary Table 4. Saturation magnetization values of MNPs, SiMNPs, CMNPs, and Ab-MNPs estimated by vibrating sample magnetometer at 25 °C.

Supplementary Table 5. Comparison of the analytical sensitivity of the SNAFIA test targeting cyclase-associated protein 1 (CAP1) protein in PBS, artificial tear fluid (ATF), and human serum solutions using analytical constants of the linear regression curve.

Supplementary Table 6. Fluorescence signal and recovery values of SNAFIA under different buffer conditions.

Supplementary Table 7. Comparison of the analytical performance of ELISA, half-SNAFIA (hSNAFIA), and SNAFIA tests targeting CAP1 protein in PBS using bioanalytical constants from a four-parameter logistic curve.

Supplementary Table 8. Comparison of the analytical sensitivity of the SNAFIA test targeting apolipoprotein E (APOE) protein in PBS, human serum, and ATF using analytical constants of the linear regression curve.

Supplementary Table 9. Comparison of the analytical performance of ELISA, hSNAFIA, and SNAFIA tests targeting APOE protein in PBS using bioanalytical constants from a four-parameter logistic curve.

Supplementary Table 10. Demographic characteristics of participants of verification cohort for clinical diagnostics application of SNAFIA.

Supplementary Notes

Supplementary Note 1. Synthesis and characterization of magnetic nanoparticles (MNPs)

Supplementary Note 2. Synthesis and characterization of polymeric nanoprobes (PNPs)

Supplementary Figures

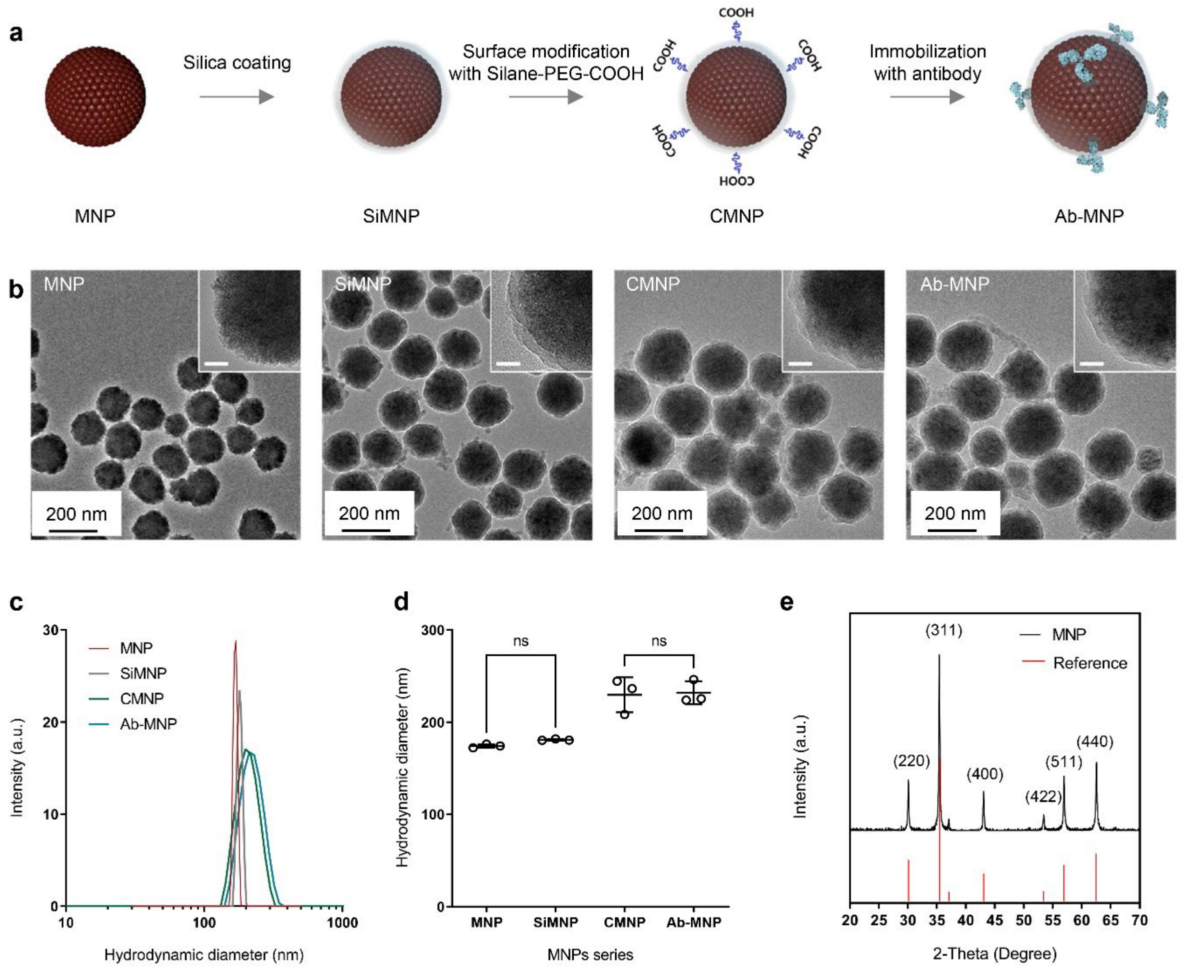

Supplementary Fig. 1 Characterization of the synthesized antibody-immobilized magnetic nanoparticles (Ab-MNPs). (a) Schematic of antibody immobilization process. (b) Transmission electron microscope (TEM) images of the magnetic nanoparticles (MNPs) series dispersed in deionized water (DW). The scale bar represents 200 nm (inset, 20 nm). The representative images were taken from different samples and repeated at least 10 times independently collection with similar results. (c) Hydrodynamic size distribution of the MNPs series dispersed in DW at a 0.1 mg/mL concentration determined by dynamic light scattering (DLS). (d) Hydrodynamic diameters of the MNPs series. (e) Powder X-ray diffraction patterns of the MNPs. A diffraction pattern of the reported Fe_3O_4 (JCPDS. 01-017-4918) is also shown. The representative graph was taken at different samples and repeated at least 3 times in independent collection with similar results. Data represent mean ± s.d. of three independent experiments (n = 3). The unit (a.u.) denotes arbitrary units. Statistical analysis was performed by multiple comparisons of Dunn's analysis of variance tests (ns, non-significant). Schematics were created with BioRender.com.

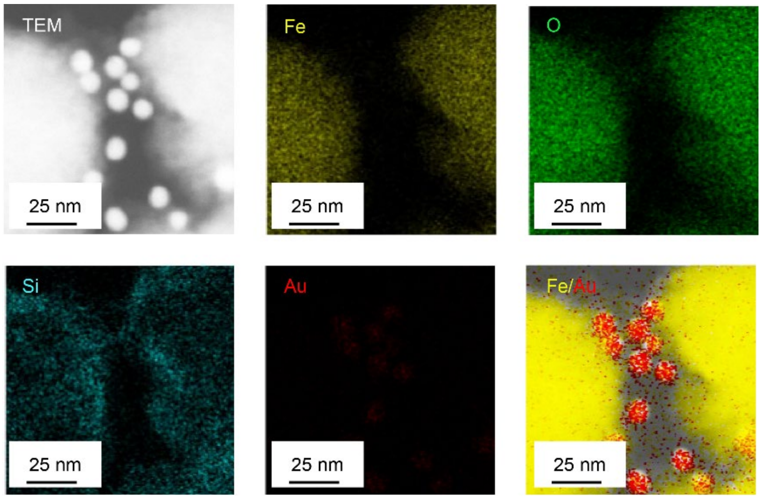

Supplementary Fig. 2 Elemental mapping images of Ab-MNPs investigated by TEM equipped with an energy-dispersive X-ray spectroscopy (EDS) detector. The elemental maps confirmed iron (Fe), oxygen (O), and silica (Si) from Ab-MNPs and gold (Au) from the immunogold localized on the surface of Ab-MNPs. Merged images of iron and gold maps were shown. The representative images were taken at different samples and repeated at least 5 times independently collection with similar results. The scale bar represents 25 nm.

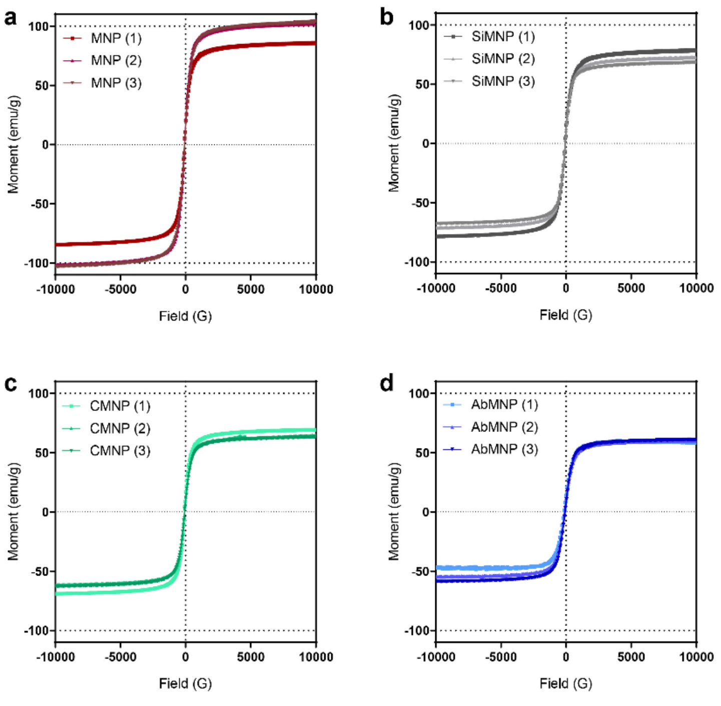

Supplementary Fig. 3 Magnetization curves of the MNPs series using vibrating sample magnetometry: (a) MNPs, (b) SiMNPs, (c) CMNPs, and (d) Ab-MNPs. All data were performed in triplicate at 25 °C (n = 3).

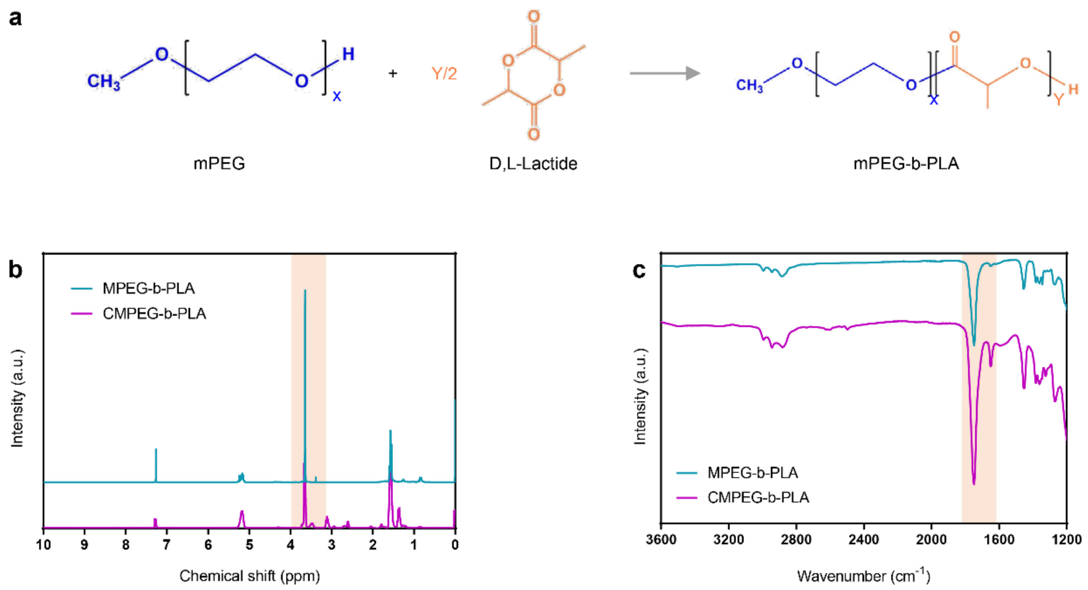

Supplementary Fig. 4 Preparation of block copolymers. (a) Schematic of synthesis of methoxy polyethylene glycol-*block*-polylactic acid (mPEG-*b*-PLA) copolymers by ring-opening polymerization. (b) ^{1}H nuclear magnetic resonance (NMR) spectra of COOH-PEG-*b*-PLA (magenta) and mPEG-*b*-PLA (turquoise). Chloroform-d was used as a solvent. The molecular weight and PLA content of the synthesized mPEG-*b*-PLA were determined with chemical shift values of 3.65 ppm (–CH_2– of PLA) and 3.38 ppm (–OCH_3– of mPEG). (c) Fourier-transform infrared spectra of COOH-PEG-*b*-PLA (magenta) and mPEG-*b*-PLA (turquoise). Their chemical structures were analyzed by the presence of characteristic peaks at 1750 cm^{-1}, indicating carbonyl stretching (C=O) of the ester in mPEG-*b*-PLA. The representative graph was estimated at different samples and repeated at least 3 times in independent collection with similar results. The unit (a.u.) denotes arbitrary units.

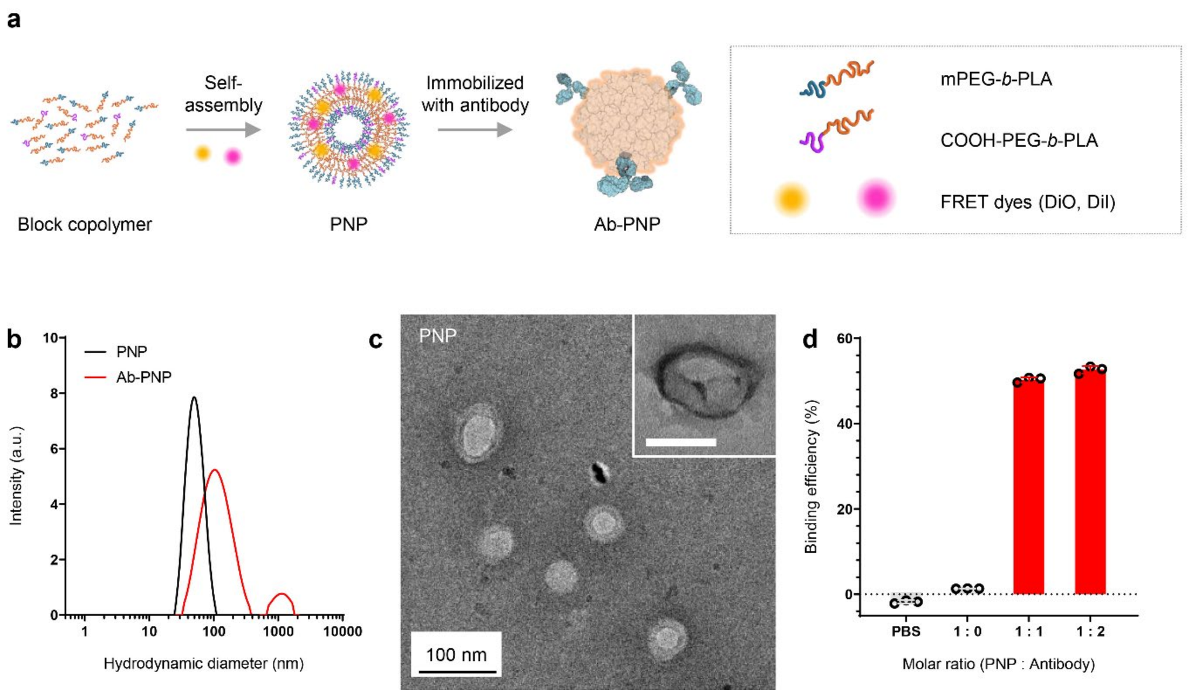

Supplementary Fig. 5 Synthetic process and characterization of antibody-immobilized polymeric nanoprobes (Ab-PNPs). (a) Schematic of Ab-PNP synthesis. mPEG-*b*-PLA, COOH-PEG-*b*-PLA, and Förster resonance energy transfer (FRET) dyes were mixed and self-assembled into PNPs. The hydrophobic FRET dyes were inserted into the hydrophobic membrane of the PNPs. Antibodies specific to the target protein were immobilized on the surface of PNPs. (b) Hydrodynamic size distribution of PNPs (black line) and Ab-PNPs (red line) by DLS. The representative graph was estimated at different samples and repeated at least 3 times in independent collection with similar results. The unit (a.u.) denotes arbitrary units. (c) TEM images of PNPs. The scale bars represent 100 nm and 20 nm (inset), respectively. The representative images were taken from different samples and repeated at least 50 times independently collection with similar results. (d) Quantification of antibody binding efficiency of Ab-PNPs prepared at different molar ratios of PNP to antibodies using bicinchoninic acid (BCA) assay. Data represent mean ± s.d. of three independent measurements ($n = 3$).

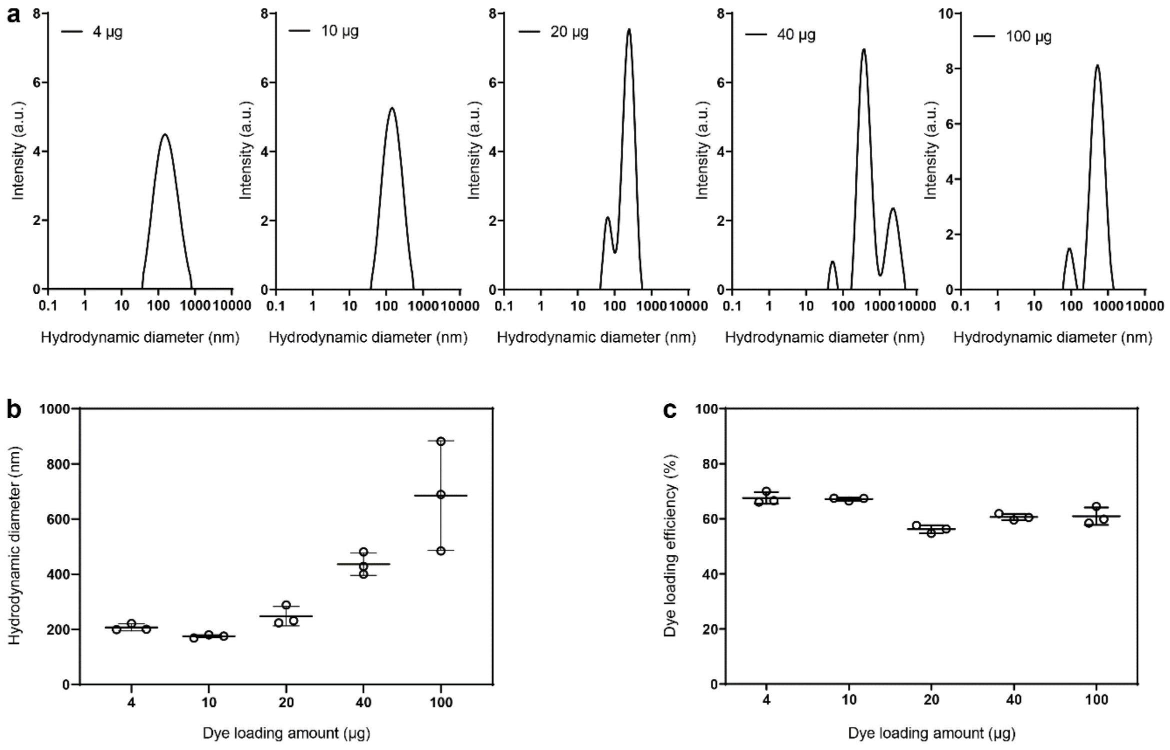

Supplementary Fig. 6 Examination of the loading amount of FRET dye. (a, b) Hydrodynamic size distribution and average size for PNPs containing different amounts of FRET dye. The representative hydrodynamic distribution graphs were obtained at different samples and repeated at least 3 times in independent collection with similar results. The unit (a.u.) denotes arbitrary units. (c) Dye loading efficiency of PNPs. Data represent mean ± s.d. of three independent experiments ($n = 3$).

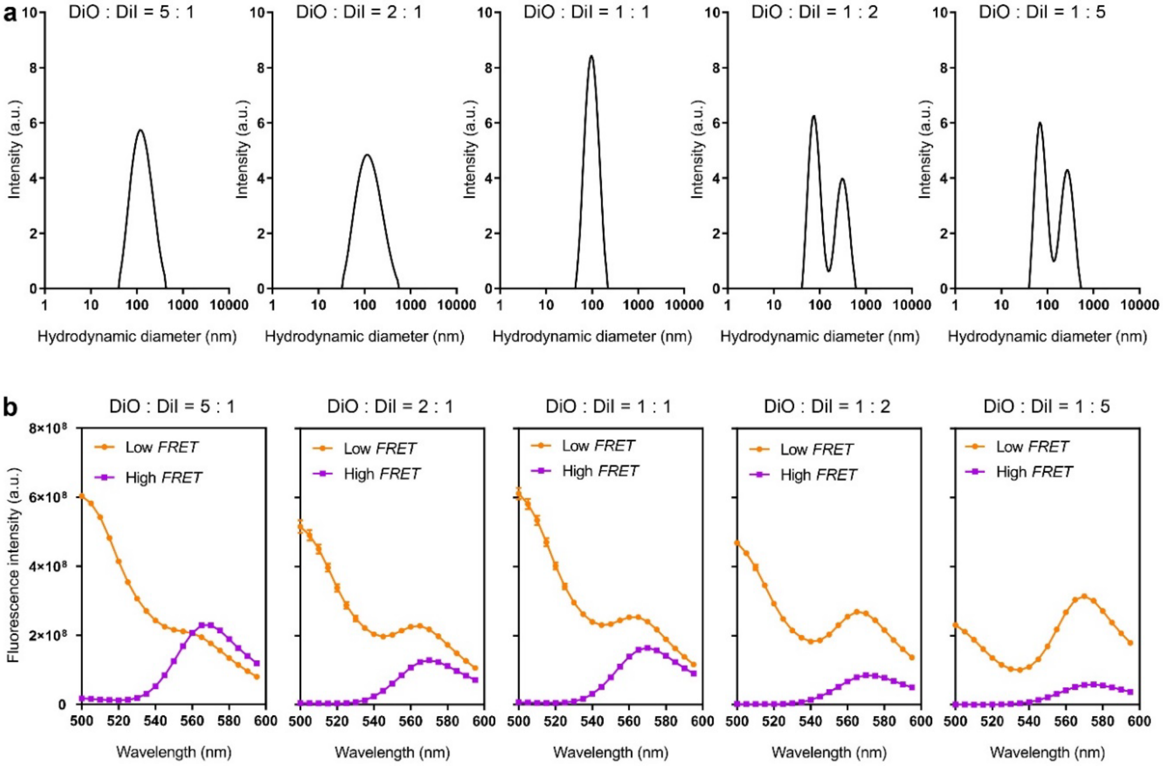

c

PNP	A	B	C	D	E
DiO : DiI	5 : 1	2 : 1	1 : 1	1 : 2	1 : 5
Hydrodynamic diameter (nm)	113.4	101.5	100.2	96.8	108.2
PDI	0.189	0.205	0.172	0.248	0.264

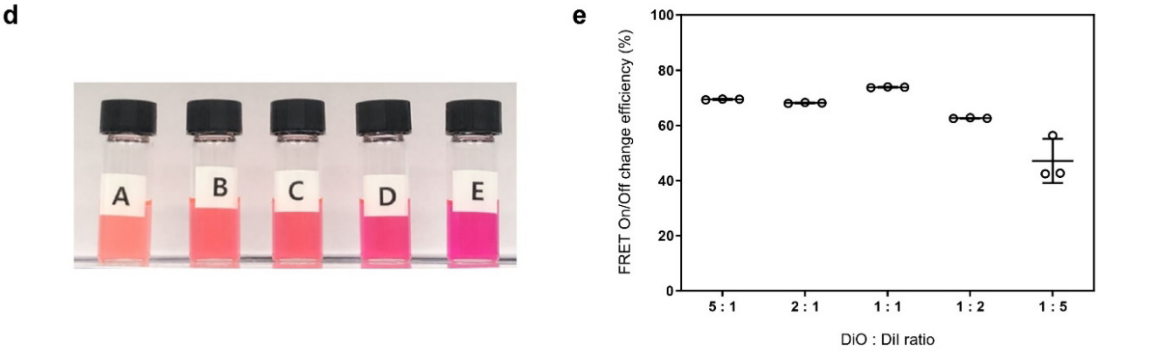

Supplementary Fig. 7 Investigation of optimal FRET dye ratios incorporated in PNPs. (a) DLS graphs of PNPs encapsulating different molar ratios of FRET dyes (DiO:DiI = 5:1, 2:1, 1:1, 1:2, and 1:5). The representative hydrodynamic distribution graphs were obtained at different samples and repeated at least 3 times in independent collection with similar results. (b) Emission fluorescence spectra of PNPs before (orange line, low FRET) and after (magenta line, high FRET) treatment with lysis buffer (excitation at 475 nm). (c) Summary of hydrodynamic diameter and

polydispersity index (PDI) values of PNPs with different ratios of FRET dyes obtained from DLS. (d) Photographs of PNP solutions containing different ratios of FRET dye. (e) Calculated FRET change efficiency of each PNPs. Fluorescent spectra and FRET change efficiency values were obtained from experiments performed in triplicate. The unit (a.u.) denotes arbitrary units. Data represent mean ± s.d. (n = 3).

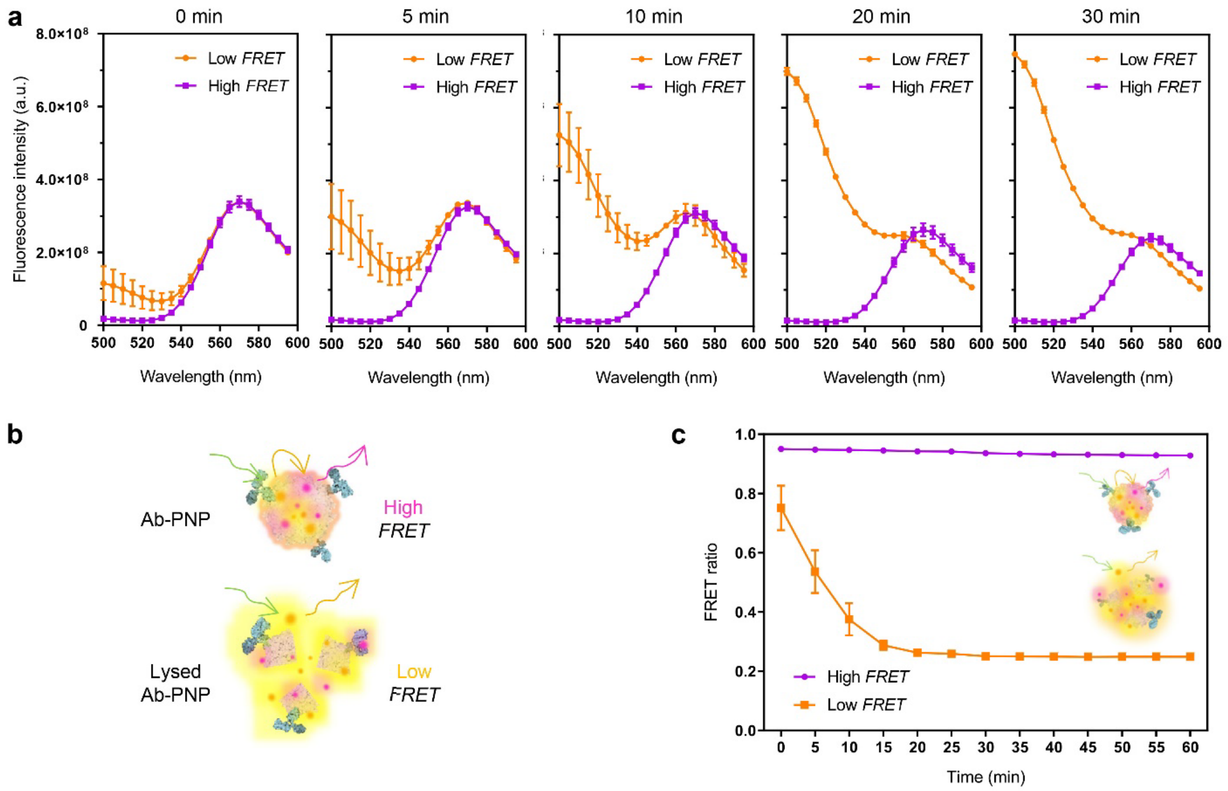

Supplementary Fig. 8 Examination of the reaction time required to induce the maximum occurrence of the high FRET to low FRET transition by PNP degradation with the addition of Triton X-100 as lysis buffer. (a) Emission fluorescence spectra of PNPs before (magenta line, high FRET) and after (orange line, low FRET) lysis buffer treatment measured at time intervals of 0, 5, 10, 20, and 30 min (excitation at 475 nm). The unit (a.u.) denotes arbitrary units. (b) Schematic of FRET signal changes of Ab-PNPs induced by treatment with TX-100 surfactant as lysis buffer. (c) Changes in the FRET ratio of PNPs over 1 hour before (high FRET) and after (low FRET) lysis buffer treatment. Data present mean ± s.d. for three independent experiments ($n = 3$).

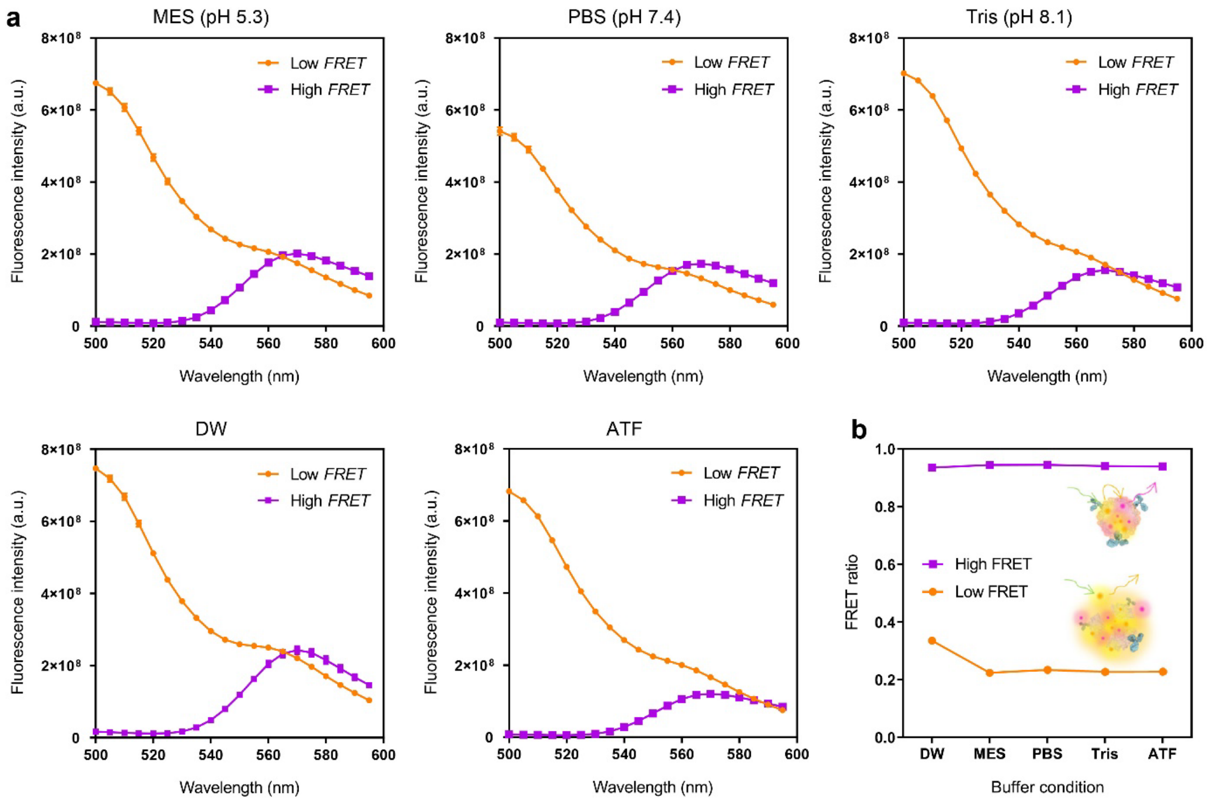

Supplementary Fig. 9 Optimization of FRET signal changes of PNPs at various buffer conditions. (a) Emission fluorescence spectra of PNPs before (magenta line, high FRET) and after (orange line, low FRET) lysis buffer treatment under various salt and pH conditions (excitation at 475 nm). The unit (a.u.) denotes arbitrary units. (b) Changes in the FRET ratio of PNPs before (high FRET) and after (low FRET) lysis buffer treatment under different salt conditions. Data present mean ± s.d. for three independent experiments (n = 3).

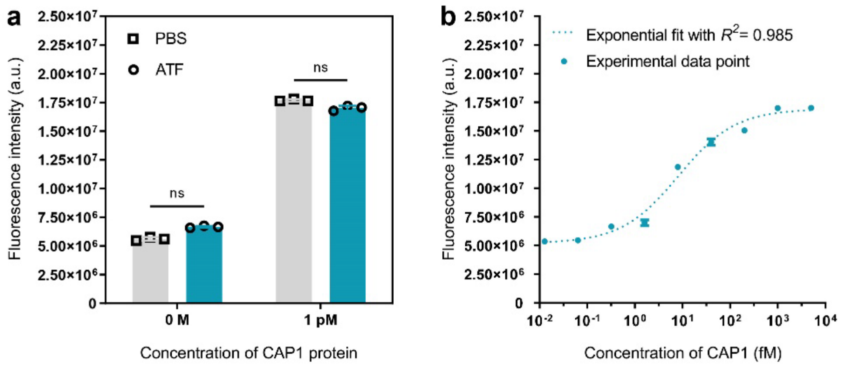

Supplementary Fig. 10 Protein marker detection efficiency of self-assembled nanoparticle-mediated amplified fluorogenic immunoassay (SNAFIA) in tear fluid. (a) Signal recovery test of SNAFIA with 0 and 1 pM of adenylyl cyclase-associated protein 1 (CAP1) protein treated in phosphate-buffered saline (PBS, grey bar) and artificial tear fluid (ATF, turquoise bar). The statistical analysis was performed by the Mann-Whitney test (ns, non-significant). (b) Sensitivity test of SNAFIA with CAP1 protein spiked into ATF. Fluorescence intensity data were fitted to a four-parameter logistic regression (dotted line). The unit (a.u.) denotes arbitrary units. Data present mean ± s.d. for three independent experiments (n = 3).

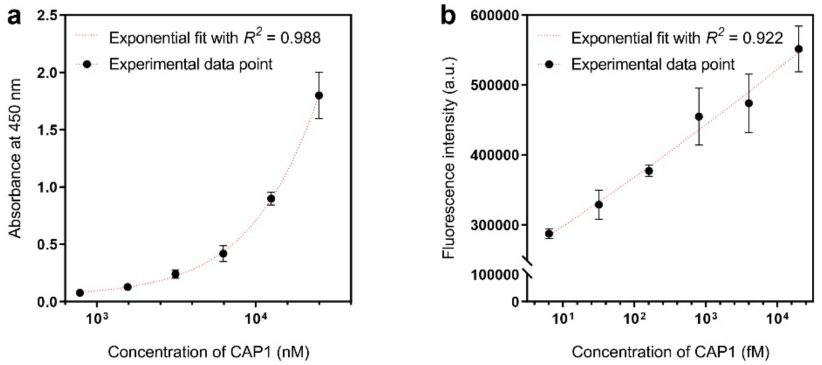

Supplementary Fig. 11 ELISA and hSNAFIA tests with different concentrations of CAP1 protein ranging from 0 to 25000 nM. CAP1 dose-responsive curves and exponential regression obtained by (a) ELISA and (b) hSNAFIA tests, respectively. The unit (a.u.) denotes arbitrary units. Data present mean ± s.d. for a duplicate in ELISA (n = 2) and triplicate experiments in hSNAFIA (n = 3).

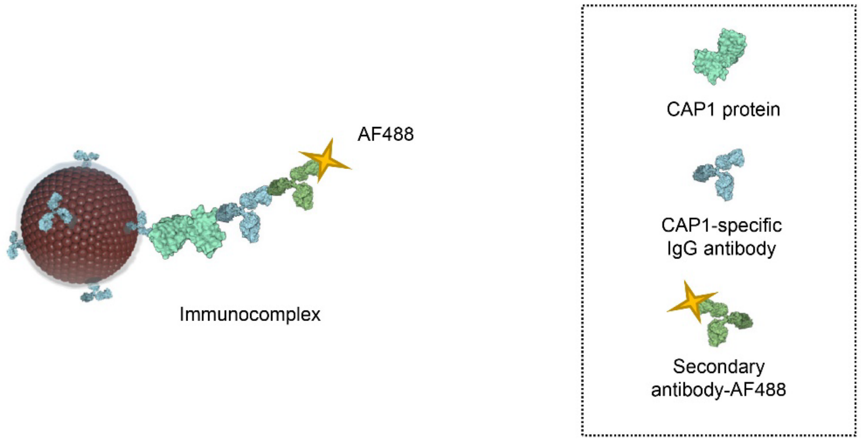

Supplementary Fig. 12 Schematic of half–SNAFIA (hSNAFIA) designed by replacing Ab-PNPs in SNAFIA with AlexaFluor®488 (AF488)-conjugated antibodies. Immunocomplexes were formed following the same protocol as for SNAFIA, with an AF488-conjugated secondary antibody to capture and label the CAP1-specific primary antibody.

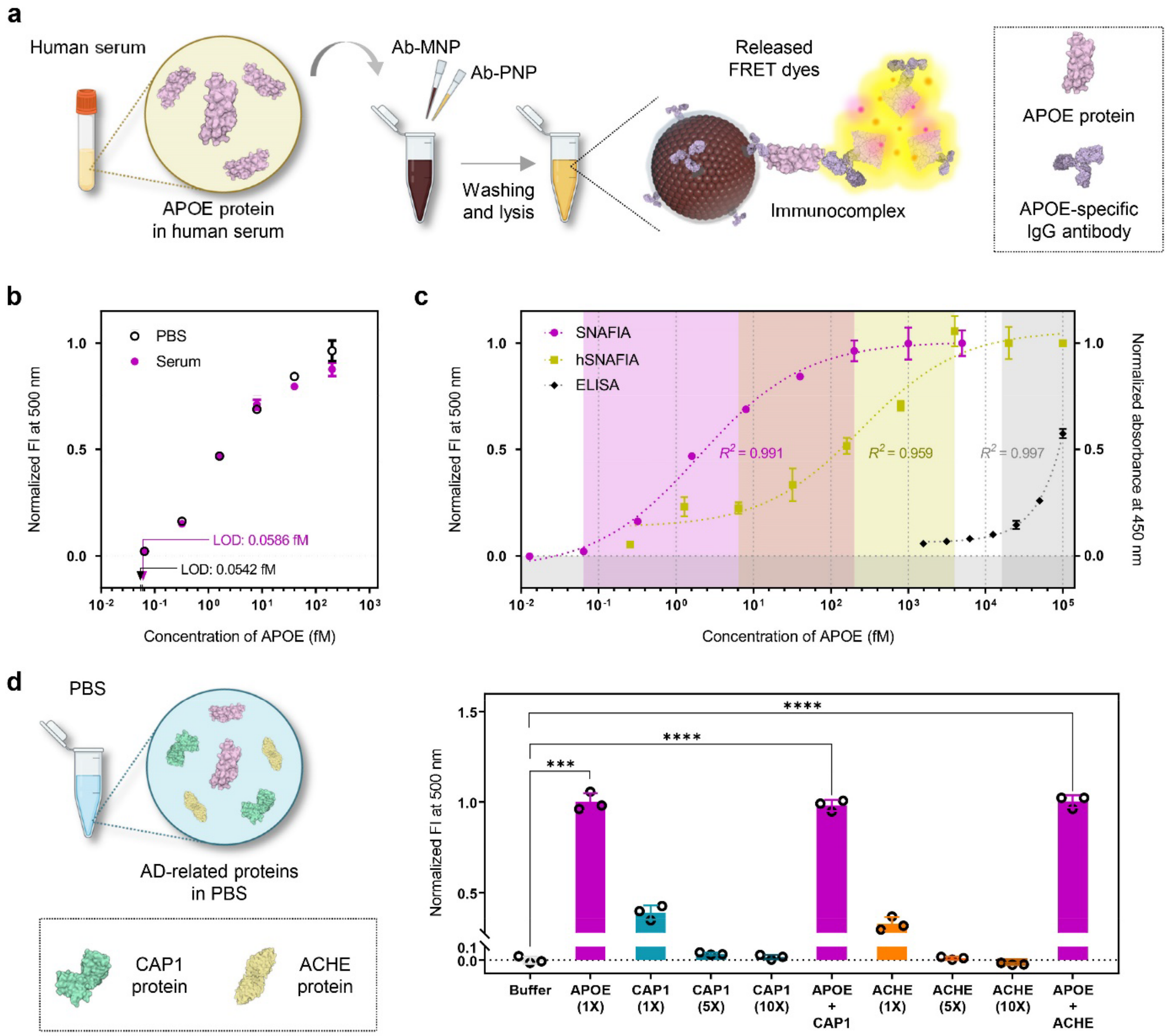

Supplementary Fig. 13 Evaluation of the SNAFIA platform toward apolipoprotein E (APOE) spiked in human serum. (a) Schematic of the SNAFIA test process for detection of APOE protein in serum. (b) Normalized fluorescence intensity (FI) of SNAFIA with increasing concentrations of APOE protein spiked in PBS (black circles) and 10% (v/v) human serum (excitation at 475 nm, emission at 500 nm, and magenta circles). The limit of detection (LOD) of SNAFIA in each condition was determined by three-sigma (3σ) and reported to be 0.0542 and 0.0586 fM, respectively. (c) Fluorescence and absorbance signal responses of the SNAFIA (magenta dots), half-SNAFIA (hSNAFIA, yellowish dots), and ELISA (black dots) tests with different concentrations of APOE protein ranging from 10^{-2} to 10^{5} fM. Normalized FI and absorbance data were fitted to the four-parameter logistic regression (dotted line). The linear dynamic range of each test is shown by the shaded area. (d) Selectivity of SNAFIA for various AD biomarkers and their mixtures in PBS. The concentrations of APOE (magenta bars), CAP1 (turquoise bars), and acetylcholinesterase (ACHE, orange bars) were varied to 1 pM (1×), 5 pM (5×), and 10 pM (10×).

Statistical analysis was performed by multiple comparisons of Brown-Forsythe and Welch one-way analysis of variance tests (****$p < 0.0001$, ***$p = 0.0002$). The measurement was performed in triplicate, and all reported values represent mean ± s.d.; $n = 3$ repeated tests. Schematics were created with BioRender.com.

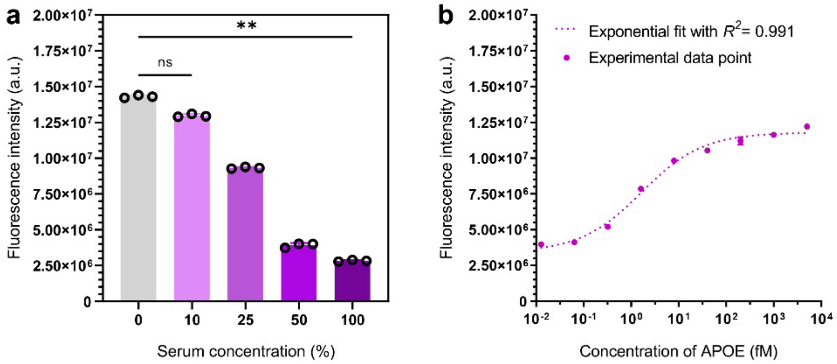

Supplementary Fig. 14 Assessment of the colloidal stability of PNPs in human serum. (a) Fluorescence intensity obtained by SNAFIA in human serum solutions prepared at 0, 10, 25, 50, and 100% (v/v) dilution in PBS. The concentration of APOE was 1 nM. The statistical analysis was performed by multiple comparisons of Dunn's analysis of variance tests (ns, non-significant, ***p* = 0.0041). (b) Sensitivity test of SNAFIA by spiking APOE protein into 10% (v/v) human serum solution. Fluorescence data were fitted to a four-parameter logistic regression (dotted line). The unit (a.u.) denotes arbitrary units. Data present mean ± s.d. for three independent experiments (*n* = 3).

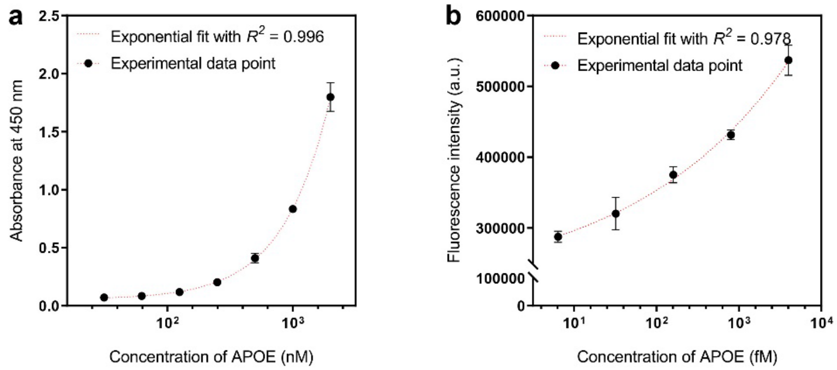

Supplementary Fig. 15 ELISA and hSNAFIA test with different concentrations of APOE protein ranging from 0 to 2000 nM. APOE dose-responsive curves and exponential regression obtained by (a) ELISA and (b) hSNAFIA tests, respectively. The unit (a.u.) denotes arbitrary units. Data present mean ± s.d. for a duplicate in ELISA (n = 2) and triplicate experiments in hSNAFIA (n = 3).

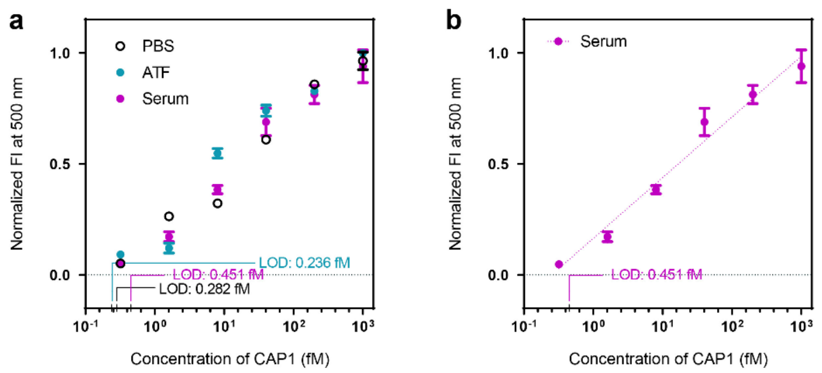

Supplementary Fig. 16 Sensitivity test of SNAFIA with increasing concentration of CAP1 protein. (a) Normalized FI of SNAFIA with CAP1 protein spiked into PBS (black circles), ATF (turquoise circles), and 10% (v/v) human serum (magenta circles) (excitation at 475 nm, emission at 500 nm). (b) Normalized FI of SNAFIA with CAP1 protein spiked in 10% (v/v) serum. The LOD of SNAFIA for PBS, serum, and ATF samples was determined by 3σ and reported to be 0.282, 0.236, and 0.451 fM, respectively. The measurement was performed in triplicate, and all reported values represent mean ± s.d.; $n = 3$ repeated tests.

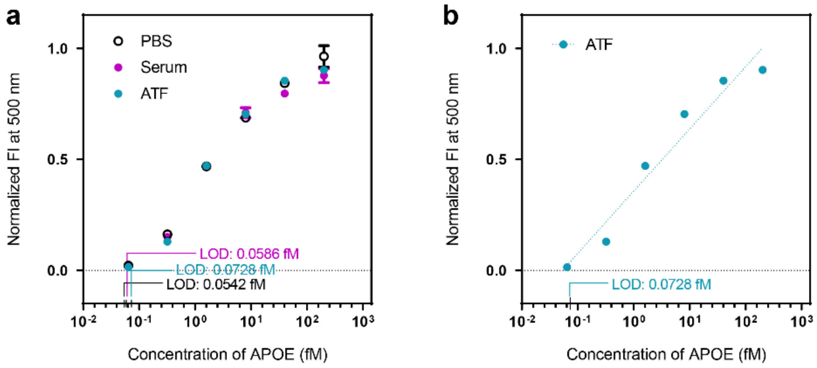

Supplementary Fig. 17 Sensitivity test of SNAFIA with increasing concentration of APOE protein. (a) Normalized FI of SNAFIA with APOE protein spiked into PBS (black circles), 10% (v/v) human serum (magenta circles), and ATF (turquoise circles) (excitation at 475 nm, emission at 500 nm). (b) Normalized FI of STNAFIA with APOE protein spiked in ATF. The LOD of SNAFIA for PBS, serum, and ATF samples was determined by 3σ and reported to be 0.0542, 0.0586, and 0.0728 fM, respectively. The measurement was performed in triplicate, and all reported values represent mean ± s.d.; $n = 3$ for independently repeated tests.

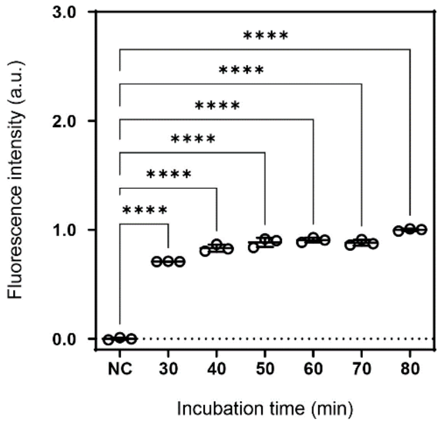

Supplementary Fig. 18 Investigation of the optimal reaction time for SNAFIA tests. Fluorescence signals were analyzed as a function of reaction time. The concentration of APOE was 1 pM. The statistical analysis was performed by ordinary one-way analysis of the variance of Brown-Forsythe and Barlett's tests (****$p < 0.0001$). The unit (a.u.) denotes arbitrary units. Data present mean ± s.d. for three independent experiments ($n = 3$).

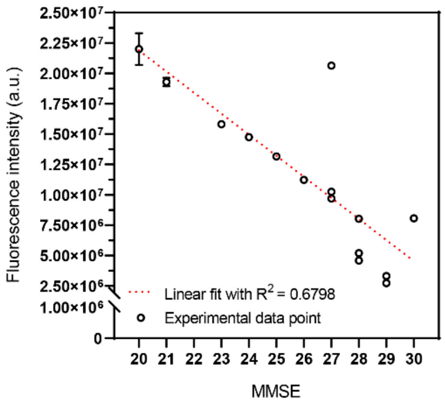

Supplementary Fig. 19 Correlation between the fluorescence intensity of SNAFIA obtained from a clinical tear sample (patient ID: 15–29) and the Mini-Mental State Examination (MMSE) score of the same subject. The scatter plot shows that the fluorescence signal obtained from the SNAFIA assay and the MMSE score is negatively correlated, with a partial correlation coefficient of 0.6798 ($p < 0.0001$). The unit (a.u.) denotes arbitrary units.

Supplementary Tables

Subject No.	Diagnosis	Age (Mean, SD)	Sex (M, %)	MMSE (Mean, SD)	CDR-SB (Mean, SD)	APOE4 positivity (%)	Amyloid-β positivity (Aβ- : Aβ+)
1	HC	64	M	28	0	0	0
2	HC	72	M	30	0	0	0
3	HC	66	F	30	0	0	0
4	HC	79	F	30	0.5	1	0
5	HC	60	M	29	0	0	0
6	HC	73	M	27	0.5	1	0
7	HC	67	M	30	0	0	0
N = 7		68.7 ± 5.9	71.4	29.1 ± 1.1	0.1 ± 0.2	28.6	7 : 0
8	MCI	61	F	25	0.5	0	1
9	MCI	83	M	22	1	0	1
10	MCI	67	M	24	1.5	1	1
11	MCI	68	M	22	1.5	1	1
12	MCI	78	F	24	2	0	1
13	MCI	66	M	27	1	0	1
14	MCI	75	M	26	2	0	1
N = 7		71.1 ± 7.2	71.4	24.3 ± 1.7[a]	1.4 ± 0.5	28.6	0 : 7
15	AD	75	M	16	10	1	1
16	AD	73	M	19	10	0	1
17	AD	64	F	19	6	1	1
18	AD	67	M	21	6	1	1
19	AD	78	F	13	10	1	1
20	AD	76	M	18	8	0	1
21	AD	80	M	9	11	0	1
N = 7		73.3 ± 5.4	71.4	16.4 ± 3.8[a, b]	8.7 ± 1.9	57.1	0 : 7

Supplementary Table 1 Demographics of participants of the discovery cohort for tear protein biomarker profiling in this study. Statistical analysis was performed by multiple comparisons of Brown-Forsythe and Welch one-way analysis of variance tests ([a]$p = 0.0002$ for the comparisons

between healthy control (HC) and each group. [b]$p = 0.004$ for the comparisons between mild cognitive impairment (MCI) and Alzheimer's disease (AD) groups). Data present mean ± s.d. ($N = 7$ for each group).

No.	DEPs		
	Gene Symbol (MCI_AD_Common_DEP)	Fold change (MCI/HC)	Fold change (AD/HC)
1	RETN	2.34	1.58
2	CST2	2.22	1.86
3	SERPINA2	2.05	1.86
4	AGR2	2.02	1.64
5	BASP1	1.96	1.63
6	CES1	1.87	1.53
7	LRRC59	1.76	1.77
8	ALCAM	1.73	1.60
9	CAP1	1.72	1.86
10	GLUL	1.71	1.69
11	NAMPT	1.70	1.77
12	PDIA3	1.68	1.84
13	HMGB1	1.66	1.82
14	HIST1H1B	1.65	1.56
15	PSMB10	1.65	2.78
16	CAMP	1.65	1.69
17	SUB1	1.64	1.66
18	APOA4	1.63	1.65
19	VAPB	1.61	1.80
20	TOP1	1.61	1.68
21	HMGA1	1.60	1.74
22	FBL	1.60	2.16
23	AARS	1.60	1.67
24	KTN1	1.60	1.78
25	HSPB1	1.59	1.92
26	LMNB2	1.59	1.72
27	DNPEP	1.58	1.81
28	PSMA4	1.57	2.60
29	UTRN	1.57	1.57
30	ARGLU1	1.57	1.68
31	MARCKS	1.57	1.51
32	LCP1	1.57	1.56
33	TMA7	1.57	1.64
34	STK10	1.57	1.54
35	FAHD2A	1.57	1.50
36	ERP29	1.56	1.72
37	MANF	1.55	1.52
38	CCDC50	1.54	1.71
39	TROVE2	1.54	1.96
40	RALY	1.54	1.67
41	UBLCP1	1.54	2.09
42	TXNDC5	1.54	1.53
43	TAF15	1.53	1.66

44	BDH2	1.53	1.61
45	PRPSAP1	1.53	1.65
46	LAGE3	1.53	1.63
47	IGHV3-20	1.53	1.55
48	ILF3	1.53	1.72
49	SERPINC1	1.52	1.59
50	DSG2	1.52	1.61
51	TLN1	1.52	1.63
52	PTBP1	1.52	1.71
53	SNRPA	1.52	1.53
54	IGHG3	1.52	1.57
55	HSPA5	1.52	1.70
56	EEA1	1.51	1.57
57	HMGN4	1.51	1.66
58	STX4	1.51	1.98
59	CORO1A	1.51	1.72
60	SNRNP70	1.51	1.82
61	TCERG1	1.50	1.60
62	ACTB	1.50	1.59
63	PDAP1	1.50	1.50
64	CLINT1	1.50	1.63
65	PLA2G2A	0.67	0.54
66	LCN1	0.67	0.58
67	B2M	0.66	0.65
68	LACRT	0.63	0.52
69	PIP	0.55	0.57
70	PSCA	0.52	0.37
71	PRELP	0.49	0.48
72	SCGB1D1	0.48	0.43
73	SCGB2A1	0.46	0.43
74	IGKV1-9	0.39	0.38
75	SMR3B	0.36	0.49

Supplementary Table 2. Differentially expressed proteins (DEPs) in tear fluid of MCI and AD compared to HC. Sixty-four proteins exhibiting fold changes greater than 1.5 classified as upregulated (red) and eleven proteins showing a fold change less than 0.67 classified as downregulated (blue). Statistical analysis was performed for each protein using a *t*-test ($p < 0.01$).

Size (nm)	MNP	SiMNP	CMNP	Ab-MNP
DLS	168.1 ± 5.9	180.6 ± 7.9	208.2 ± 36.3	223.8 ± 39.9
TEM	151.8 ± 12.8	160.7 ± 18.0	183.2 ± 17.7	187.5 ± 5.2

Supplementary Table 3 Size of MNPs, SiMNPs, CMNPs, and Ab-MNPs estimated by DLS and TEM image-based analysis. Data present mean ± s.d. for three independent experiments (n = 3).

Saturation Magnetization (emu/g)	MNP	SiMNP	CMNP	Ab-MNP
Cycle 1	90.1	78.5	69.6	56.2
Cycle 2	107	72.4	65.9	60.1
Cycle 3	106	70.2	64.7	59.7
Average	101	73.7	66.8	58.6

Supplementary Table 4 Saturation magnetization values of MNPs, SiMNPs, CMNPs, and Ab-MNPs estimated by vibrating sample magnetometer at 25 °C.

Parameter	PBS	ATF	Human serum
Slope	0.271	0.278	0.273
Y-intercept	0.172	0.204	0.165
X-intercept	-0.635	-0.728	-0.603
R squared	0.975	0.944	0.968
Limit of detection (fM)	0.283	0.236	0.451
Limit of quantification (fM)	0.447	0.406	0.823

Supplementary Table 5 Comparison of the analytical sensitivity of the SNAFIA test targeting cyclase-associated protein 1 (CAP1) protein in PBS, artificial tear fluid (ATF), and human serum solutions using analytical constants of the linear regression curve. The limit of detection (LOD) and limit of quantification (LOQ) were based on the three-sigma (3σ) and ten-sigma (10σ) methods, respectively.

Fluorescence signal (a.u.)	PBS	ATF	Recovery (%)
0 M	439000	666000	152
1 M	1770000	17000000	96.0

Supplementary Table 6 Fluorescence signal and recovery values of SNAFIA under different buffer conditions. The unit (a.u.) denotes arbitrary units.

Parameter	ELISA	hSNAFIA	SNAFIA
Bottom	0.0916	1.15	-0.0498
Top	0.981	-0.0585	1.08
LogEC50	7.76	2.35	1.29
HillSlope	1.51	0.375	0.528

Supplementary Table 7 Comparison of the analytical performance of ELISA, half-SNAFIA (hSNAFIA), and SNAFIA tests targeting CAP1 protein in PBS using bioanalytical constants from a four-parameter logistic curve.

Parameter	PBS	Human serum	ATF
Slope	0.285	0.265	0.280
Y-intercept	0.367	0.357	0.357
X-intercept	-1.29	-1.35	-1.28
R squared	0.977	0.949	0.953
Limit of detection (fM)	0.0542	0.0586	0.0728
Limit of quantification (fM)	0.0609	0.111	0.154

Supplementary Table 8 Comparison of the analytical sensitivity of the SNAFIA test targeting apolipoprotein E (APOE) protein in PBS, human serum, and ATF using analytical constants of the linear regression curve. The LOD and LOQ were based on the 3σ and 10σ methods, respectively.

Parameter	ELISA	hSNAFIA	SNAFIA
Bottom	0.0642	0.134	-0.0631
Top	~ 883	1.06	1.01
LogEC50	~ 7.40	2.40	0.306
HillSlope	~ 1.35	0.680	0.644

Supplementary Table 9 Comparison of the analytical performance of ELISA, hSNAFIA, and SNAFIA tests targeting APOE protein in PBS using bioanalytical constants from a four-parameter logistic curve.

Subject No.	Diagnosis	Age (Mean, SD)	Sex (M, %)	MMSE (Mean, SD)	CDR-SB (Mean, SD)	APOE4 positivity (%)	Amyloid-β positivity (Aβ- : Aβ+)
1	HC	79	M	25	2	0	1
2	HC	73	F	-	-	-	-
3	HC	77	M	23	1.5	0	0
4	HC	84	F	27	1.5	0	0
5	HC	66	M	29	0.5	1	0
6	HC	76	M	28	1.5	0	0
7	HC	64	F	30	0.5	1	1
8	HC	50	F	30	0.5	0	0
9	HC	77	M	29	0.5	0	0
10	HC	77	F	29	0.5	1	1
11	HC	68	F	26	0.5	0	0
12	HC	73	M	30	0	0	-
13	HC	74	M	30	0.5	0	1
14	HC	63	F	28	1.5	1	0
N = 14		71.5 ± 8.3	50.0	28.0 ± 2.1	0.9 ± 0.6	28.6	8 : 4
15	MCI	83	M	28	1	0	1
16	MCI	72	M	20	10	1	1
17	MCI	77	M	29	1	0	-
18	MCI	64	M	25	2.5	1	1
19	MCI	81	M	26	2	0	0
20	MCI	74	M	27	1.5	0	1
21	MCI	83	M	23	1.5	1	1
22	MCI	69	F	30	1	0	0
23	MCI	81	M	28	1.5	0	1
24	MCI	79	M	28	1.5	0	0
25	MCI	79	M	27	1	0	1
26	MCI	78	F	26	1	1	1
27	MCI	62	F	27	3.5	-	1
28	MCI	63	F	24	2	0	0
29	MCI	72	M	29	1	0	-

N = 15		74.5 ± 7.0	73.3	26.5 ± 2.6	2.1 ± 2.2	26.7	4 : 9
30	AD	79	F	8	4	1	0
31	AD	81	F	20	0.5	-	0
32	AD	89	M	22	5	1	1
33	AD	83	F	20	10	-	0
34	AD	90	F	13	10	0	-
35	AD	89	F	12	9.5	0	1
36	AD	87	F	7	11	0	1
37	AD	78	F	17	6	1	1
38	AD	90	M	22	8	-	-
39	AD	83	F	20	3.5	1	1
N = 10		84.9 ± 4.4	20.0	16.1 ± 5.4[a, b]	6.8 ± 3.3	40.0	3 : 5

Supplementary Table 10 Demographic characteristics of participants of verification cohort for clinical diagnostics application of SNAFIA. Statistical analysis was performed by multiple comparisons of Brown-Forsythe and Welch one-way analysis of variance tests ([a]$p < 0.0001$ for the comparisons between HC and AD groups. [b]$p = 0.0003$ for the comparisons between MCI and AD groups). Data present mean ± s.d. ($N = 14$, 15, and 10 for each group).

Supplementary Notes

1. Synthesis and characterization of magnetic nanoparticles (MNPs)

To construct a magnetic separation system, we synthesized MNPs using a solvothermal method[1, 2] to efficiently capture target proteins in human body fluids. To conjugate antibodies to the MNPs' surface, silica-coated MNPs (SiMNPs) and carboxylated polyethylene glycol (PEG)-functionalized MNPs (CMNPs) were sequentially prepared via the sol-gel technique. These silica and PEG shells not only protect the MNPs' surface from oxidation in air but also allow them to maintain stable colloidal dispersion in an aqueous solution for an extended period of time. We then prepared Ab-MNPs by coupling between the carboxyl group of CMNPs and the amine group of antibodies using 1-ethyl-3-(3-(dimethylamino)-propyl) carbodiimide and *N*-hydroxysulfosuccinimide (EDC/sulfo-NHS) chemistry (**Supplementary Fig. 1a**). To investigate the effect of surface modification on morphology and size, MNPs whose surfaces were modified with silica, PEG, and antibodies, respectively, were compared to intact MNPs using transmission electron microscopy (TEM) and dynamic light scattering (DLS) (**Supplementary Figs. 1b-d**). Representative TEM images showed no noticeable changes in the size or spherical morphology of the particles. In the TEM images, we also observed relatively monodisperse particles with a thin polymer coating (10 nm thick) on the surface and a gradual increase in the size of the MNPs in the range of 150–190 nm after a series of surface functionalizations. The average hydrodynamic sizes of MNPs, SiMNPs, CMNPs, and Ab-MNPs were measured to be 170–230 nm, which is consistent with the sizes observed in TEM, considering the hydrated polymer and biomolecular coating (**Supplementary Table 3**). The X-ray diffraction pattern of the as-prepared MNPs confirmed their presence in the inverse cubic spinel phase of iron oxide (Fe_3O_4) (JCPDS no. 01-017-4918) (**Supplementary Fig. 1e**).

2. Synthesis and characterization of polymeric nanoprobes (PNPs)

To prepare the PNPs, methoxy PEG-*block*-polylactic acid (mPEG-*b*-PLA) copolymers with an *f*-value of 0.22 (defined as the molar ratio of a hydrophilic segment to total polymer unit) were first synthesized using tin (II)-ethyl hexanoate-catalyzed ring-opening polymerization (Supplementary **Fig. 4**). The high hydrophobicity of PLA block belonging to aliphatic polyesters induces Förster resonance energy transfer (FRET) dye to be easily loaded[3]. After adding FRET dyes, thin-film hydration and tip sonication were used to induce the copolymers to assemble into PNPs with membrane structures. After removing free dyes through successive dialysis, the target protein-specific antibody was immobilized on the surface of PNPs using EDC/sulfo-NHS chemistry and purified by dialysis again. The resulting product was referred to as Ab-PNPs (**Supplementary Fig. 5a**). DLS showed a 21.4% increase in size upon antibody binding (**Supplementary Fig. 5b**). Consisting with the DLS results, TEM images confirmed that the prepared Ab-PNPs (39.7 nm) had a slight increase in size compared to PNPs (48.2 nm) and were distributed as spherical nanoparticles with relatively uniform size and monolayers (**Fig. 3f and Supplementary Fig. 5c**). We also analyzed the binding efficiency of the antibody to PNPs using the bicinchoninic acid assay, showing an antibody binding efficiency of over 50% (**Supplementary Fig. 5d**).

Supplementary References

1. Kang B. et al. Serially ordered magnetization of nanoclusters via control of various transition metal dopants for the multifractionation of cells in microfluidic magnetophoresis devices. *Anal. Chem.* **88**, 1078-1082 (2016).

2. Lee H. et al. Rapid visible detection of African swine fever virus using hybridization chain reaction-sensitized magnetic nanoclusters and affinity chromatography. *Small* **19**, 2207117 (2023).

3. Ghasemi R. et al. mPEG-PLA and PLA-PEG-PLA nanoparticles as new carriers for delivery of recombinant human Growth Hormone (rhGH). *Sci. Rep.* **8**, 9854 (2018).
