# Supplementary material for: Amplified fluorogenic immunoassay for early diagnosis and monitoring of Alzheimer’s disease from tear fluid
Source: Nat Commun. 2023 Dec 9;14:8153. doi: 10.1038/s41467-023-43995-5 (PMC10710446; doi:10.1038/s41467-023-43995-5)
Supplement: Supplementary file 3 — Description of Additional Supplementary Information [file 41467_2023_43995_MOESM3_ESM.pdf]

Description of Additional Supplementary Files

Supplementary Data

Supplementary Data 1. List of total proteins identified from proteomic analysis conducted in this study.
