# Supplementary material for: Amplified fluorogenic immunoassay for early diagnosis and monitoring of Alzheimer’s disease from tear fluid
Source: Nat Commun. 2023 Dec 9;14:8153. doi: 10.1038/s41467-023-43995-5 (PMC10710446; doi:10.1038/s41467-023-43995-5)
Supplement: Supplementary file 5 — Reporting Summary [file 41467_2023_43995_MOESM5_ESM.pdf]

nature portfolio

Corresponding author(s): s. Haam, Y. w. Ji, H. Cho

Last updated by author(s): Oct 25, 2023

Reporting Summary

Nature Portfolio wishes to improve the reproducibility of the work that we publish. This form provides structure for consistency and transparency in reporting. For further information on Nature Portfolio policies, see our Editorial Policies and the Editorial Policy Checklist.

Statistics

For all statistical analyses, confirm that the following items are present in the figure legend, table legend, main text, or Methods section.

n/a	Confirmed	
☐	☒	The exact sample size (*n*) for each experimental group/condition, given as a discrete number and unit of measurement
☐	☒	A statement on whether measurements were taken from distinct samples or whether the same sample was measured repeatedly
☐	☒	The statistical test(s) used AND whether they are one- or two-sided<br>*Only common tests should be described solely by name; describe more complex techniques in the Methods section.*
☒	☐	A description of all covariates tested
☒	☐	A description of any assumptions or corrections, such as tests of normality and adjustment for multiple comparisons
☐	☒	A full description of the statistical parameters including central tendency (e.g. means) or other basic estimates (e.g. regression coefficient) AND variation (e.g. standard deviation) or associated estimates of uncertainty (e.g. confidence intervals)
☐	☒	For null hypothesis testing, the test statistic (e.g. *F*, *t*, *r*) with confidence intervals, effect sizes, degrees of freedom and *P* value noted<br>*Give P values as exact values whenever suitable.*
☒	☐	For Bayesian analysis, information on the choice of priors and Markov chain Monte Carlo settings
☒	☐	For hierarchical and complex designs, identification of the appropriate level for tests and full reporting of outcomes
☐	☒	Estimates of effect sizes (e.g. Cohen's *d*, Pearson's *r*), indicating how they were calculated

Our web collection on statistics for biologists contains articles on many of the points above.

Software and code

Policy information about availability of computer code

Data collection	Protein-protein interactome information: STRING public database (v11.5)<br>Network model: Cytoscape software (v3.7.2)<br>MS/MS spectra: Proteome Discoverer software (v2.1)<br>TEM images: DigitalMicrograph (v3.0)<br>EDS mapping: AZtec software (v6.0)<br>XRD pattern processing: JADE (v5.0)<br>VSM magnetization curves: Lake Shore IDEAS VSM (v3.6)<br>Fluorescence and absorbance: SoftMax Pro 7.0.3<br>DLS analysis: ELSZ-2000<br>1H-NMR and FT-IR specra: TopSpin 3.5 and Spectrum (v10.3.6)
Data analysis	All graphs (diffraction patterns, magnetization curves, fluorescence spectra, fluorescence intensity) were drawn using GraphPad Prism 9 (v9.0.0). Diagnostic performance (e.g., ROC curve, LOD) and statistical analysis (Pearson's coefficient, p-values) of SNAFIA were performed using SPSS 23 (v17.0.2) and GraphPad Prism 9. Image J (v23) was used for image-based nanoparticle size measurement.

For manuscripts utilizing custom algorithms or software that are central to the research but not yet described in published literature, software must be made available to editors and reviewers. We strongly encourage code deposition in a community repository (e.g. GitHub). See the Nature Portfolio guidelines for submitting code & software for further information.

Data

Policy information about availability of data

All manuscripts must include a data availability statement. This statement should provide the following information, where applicable:

- Accession codes, unique identifiers, or web links for publicly available datasets
- A description of any restrictions on data availability
- For clinical datasets or third party data, please ensure that the statement adheres to our policy

All quantified proteins are provided in the Supplementary Data file. The mass spectrometry proteomics data have been deposited to the ProteomeXchange Consortium via the PRIDE repository with the dataset identifier (PXD042142). The main data supporting the findings of this study are available in the paper and Supplementary Information. All raw and analyzed datasets generated during this study are provided as a Source Data file. Source Data are provided with this paper.

Research involving human participants, their data, or biological material

Policy information about studies with human participants or human data. See also policy information about sex, gender (identity/presentation), and sexual orientation and race, ethnicity and racism.

Reporting on sex and gender	We used the term biological sex based on each participant's study consent form and hospital-based information. Detailed demographic information of participants is provided in Supplementary Tables 1 and 10.
Reporting on race, ethnicity, or other socially relevant groupings	All participants were Asian and domestic population (South Korea). We did not use any socially relevant variables.
Population characteristics	Clinical diagnoses for brain and ocular diseases were made by consensus of one neurologist and one ophthalmologist, respectively. All participants had no ophthalmological disease except for cataracts. We excluded patients with dry eye disease; patients with any ocular history, even in the other eye, such as ocular surgery, ocular injury, ocular infection, allergy, ocular inflammation (e.g., uveitis), glaucoma, or retinal diseases; patients using topical eye drops other than artificial tears or contact lens; and patients with systemic diseases, including autoimmune disease, diabetes, and vascular disease. Participants underwent the neuropsychological test battery and brain imaging, including 18F-florbetaben positron emission tomography scans. We selected Alzheimer's disease (AD) patients who fulfilled the diagnostic criteria for "Probable AD dementia with evidence of the AD's pathophysiological process" and mild cognitive impairment (MCI) patients who fulfilled the criteria for "MCI due to AD with intermediate or high likelihood" as outlined by the US National Institute on Aging Alzheimer's Association.
Recruitment	This study was conducted in accordance with the ethical principles specified in the Declaration of Helsinki and Good Clinical Practice Guidelines. Approval was obtained by the Institutional Review Board of Yonsei University College of Medicine, and written informed consent was obtained from each patient prior to study entry. Participants were prospectively recruited from the Memory Disorder Clinic at Gangnam Severance Hospital.
Ethics oversight	This study was approved by the Institutional Ethical Review Boards of Yonsei University College of Medicine (Seoul, South Korea; IRB No. 3-2018-0156).

Note that full information on the approval of the study protocol must also be provided in the manuscript.

Field-specific reporting

Please select the one below that is the best fit for your research. If you are not sure, read the appropriate sections before making your selection.

☒ Life sciences ☐ Behavioural & social sciences ☐ Ecological, evolutionary & environmental sciences

For a reference copy of the document with all sections, see nature.com/documents/nr-reporting-summary-flat.pdf

Life sciences study design

All studies must disclose on these points even when the disclosure is negative.

Sample size	Since the tear biomarker profiling (n=7 per group) showed significant differences in tear proteins expressed among groups, an a priori power analysis was performed. As a result, a total of 33 samples from three groups were subjected to an analysis of variance (ANOVA) F-test, with a significance level (alpha) of 0.05 and a power of 95%. For the clinical tests of SNAFIA using the tear biomarker candidate, we aimed to recruit at least 36 tear samples, considering a 30% dropout rate due to insufficient tear volume, and ultimately used 39 tear samples. We also conducted a post hoc power analysis to confirm the sample size at the end of the study.
Data exclusions	Participants who gave informed consent to participate in the study were excluded from the SNAFIA tests if the amount of tear fluid collected was insufficient for proteomic analysis.
Replication	All experimental replication attempts were successful, and we will provide the raw proteome profiling data in the Pride database. Each experiment was independently replicated at least twice to support all claims in the paper.

Randomization	Our study does not involve the development of therapeutic agents, thus randomization is unnecessary.
Blinding	In the discovery and verification phases, participants who tested proteomic and SNAFIA analysis were blinded.

Reporting for specific materials, systems and methods

We require information from authors about some types of materials, experimental systems and methods used in many studies. Here, indicate whether each material, system or method listed is relevant to your study. If you are not sure if a list item applies to your research, read the appropriate section before selecting a response.

Materials & experimental systems

n/a	Involved in the study
☐	☒ Antibodies
☒	☐ Eukaryotic cell lines
☒	☐ Palaeontology and archaeology
☒	☐ Animals and other organisms
☒	☐ Clinical data
☒	☐ Dual use research of concern
☒	☐ Plants

Methods

n/a	Involved in the study
☒	☐ ChIP-seq
☒	☐ Flow cytometry
☒	☐ MRI-based neuroimaging

Antibodies

Antibodies used	Human Apo E Antibody Pair (Abcam, cat. no. ab244096) antibodies are rabbit monoclonal and primary antibodies.<br>Adenylate Cyclase 1 Rabbit anti-Human Polyclonal Antibody (LSBio, cat. no. LS-C411294, lot no. 190124) and Adenylate Cyclase 1 Rabbit anti-Human Polyclonal Antibody (LSBio, cat. no. LS-C381814, lot no. 182395) are primary antibodies.<br>ABflo 488-conjugated Goat Anti-Rabbit IgG (H+L) (ABclonal, cat.no. AS053) is secondary antibodies.
Validation	All antibodies used in the study were tested for proper performance and functionality according to the supplier's established quality-control testing criteria.<br>https://www.abcam.com/products/elisa/human-apo-e-antibody-pair-bsa-and-azide-free-ab244096.html;<br>https://www.lsbio.com/antibodies/adcy1-antibody-adenylate-cyclase-1-antibody-wb-western-ls-c411294/423659;<br>https://www.lsbio.com/antibodies/adcy1-antibody-adenylate-cyclase-1-antibody-200-280-aa-internal-elisa-ihc-wb-western-ls-c381814/393915;<br>https://abclonal.com/catalog-antibodies/AlexaFluor488conjugatedGoatAntiRabbitIgGHL/AS053.
